# Mapping the immunological battlefield in gastric cancer: prognostic implications of an immune gene expression signature

**DOI:** 10.1007/s12672-023-00834-9

**Published:** 2023-11-24

**Authors:** Xianhong Meng, Daxiu Wang, Xueying Sun, Jiangfeng Yuan, Jiwu Han

**Affiliations:** The Fourth Affiliated Hospital of Harbin Medical University, Yiyuan Street No. 37, Harbin, Heilongjiang Province 150001 China

**Keywords:** Gastric cancer, Immune-related genes, Differential expression analysis, Prognostic index, Bioinformatic analysis, Weighted gene coexpression network analysis

## Abstract

**Background:**

Gastric cancer (GC) is a heterogeneous malignancy with variable clinical outcomes. The immune system has been implicated in GC development and progression, highlighting the importance of immune-related gene expression patterns and their prognostic significance.

**Objective:**

This study aimed to identify differentially expressed immune-related genes (DEIRGs) and establish a prognostic index for GC patients using comprehensive bioinformatic analyses.

**Methods:**

We integrated RNA sequencing data from multiple databases and identified DEIRGs by overlapping differentially expressed genes with immune-related genes. Functional enrichment analysis was performed to uncover the biological processes and signaling pathways associated with DEIRGs. We conducted a Weighted Gene Co-expression Network Analysis (WGCNA) to identify key gene modules related to with GC. Cox regression analysis was conducted to determine independent prognostic DEIRGs for overall survival prediction. Based on these findings, we developed an immune-related gene prognostic index (IRGPI) based on these findings. The prognostic value of the IRGPI was validated using survival analysis and an independent validation cohort. Functional enrichment analysis, gene mutation analysis, and immune cell profiling were performed to gain insights into the biological functions and immune characteristics associated with the IRGPI-based subgroups.

**Results:**

We identified 493 DEIRGs significantly enriched in immune-related biological processes and signaling pathways associated with GC. WGCNA analysis revealed a significant module (turquoise module) associated with GC, revealing potential therapeutic targets. Cox regression analysis identified RNASE2, CGB5, CTLA4, and DUSP1 as independent prognostic DEIRGs. The IRGPI, incorporating the expression levels of these genes, demonstrated significant prognostic value in predicting overall survival. The IRGPI-based subgroups exhibited distinct biological functions, genetic alterations, and immune cell compositions.

**Conclusion:**

Our study identified DEIRGs and established a prognostic index (IRGPI) for GC patients. The IRGPI exhibited promising prognostic potential and provided insights into GC tumor biology and immune characteristics. These findings have implications for guiding therapeutic strategies.

## Introduction

Immune checkpoint inhibitor (ICI) therapy has revolutionized cancer treatment by significantly improving patient survival rates [1]. Immune checkpoint inhibitors, such as anti-PD-1 and anti-CTLA-4 antibodies, block the interaction between these checkpoints and their ligands, effectively releasing the brakes on the immune system [2]. This unleashes a potent immune response against cancer cells, leading to tumor regression and improved patient outcomes. Clinical studies have shown promising results in the treatment of advanced gastric cancer, particularly in patients whose tumors express high levels of programmed death-ligand 1 (PD-L1) protein [3, 4]. Anti-PD-1 antibodies such as pembrolizumab have demonstrated improved overall survival compared to chemotherapy in advanced gastric cancer patients in the Phase 3 KEYNOTE-061 trial [5], with an objective response rate of 11.6% in the Phase 2 Clinical KEYNOTE-059 Trial [6]. The 12-month follow-up of the Nivolumab combined with chemotherapy in gastric cancer trials has demonstrated superior overall survival rates compared to chemotherapy alone. Based on these results, Nivolumab combined with chemotherapy has now been approved as a first-line treatment option for patients with gastric cancer in many countries [6]. Nivolumab restores the functionality of anti-tumor T cells, while ipilimumab (a CTLA-4, cytotoxic T-lymphocyte antigen-4 inhibitor) induces responses in newly generated anti-tumor T cells. These agents exhibit complementary mechanisms of action. The combination therapy of nivolumab and ipilimumab has demonstrated clinically significant anti-tumor activity and manageable safety in patients with advanced gastric and esophageal cancer [7]. While immune checkpoint inhibitor (ICI) therapy has demonstrated remarkable efficacy in some cancer patients, a significant limitation is the low response rate observed in a subset of patients [8]. Despite the potential for long-lasting responses, only a fraction of patients experience a significant and durable benefit from ICI treatment [9].

Identifying the Immune-Related Gene Prognostic Index (IRGPI) can allow for the personalized immunotherapy of cancer patients. This index allows medical professionals to tailor treatments according to the individual's immunogenic profile, optimizing therapeutic response rates and minimizing adverse effects. Furthermore, the Immune-Related Gene Prognostic Index can serve as a valuable tool in clinical trials, helping identify patients more likely to benefit from immunotherapies or other targeted interventions [10]. The IRGPI has been investigated by many researchers in various types of cancer. It has been shown in the literature that IRGPI can provide a basis for personalized immunotherapy, guiding treatment selection and optimizing treatment response. For instance, in colorectal cancer research, integrating clinical and molecular information to establish IRGPI enables the prediction of patients' prognosis and immune therapy response [11]. In lung cancer research, identifying high and low-risk groups based on IRGPI can guide individualized treatment plan selection for patients [12].

The present research aimed to investigate the immune-related characteristics of gastric cancer and evaluate the prognostic value and potential clinical implications of an immune-related gene prognostic index (IRGPI). Firstly, IRGPI was constructed using multivariate Cox regression analysis and validated using survival curves. More importantly, we performed gene set enrichment analysis and gene mutation analysis to study different IRGPI subgroups, assessed immune cell infiltration using the CIBERSORT algorithm, and analyzed the distribution of immune subtypes in different IRGPI subgroups. Furthermore, the potential clinical efficacy of immunotherapy and the prognostic predictive value of IRGPI were evaluated using various methods such as the TIDE algorithm and time-dependent ROC curve analysis.

## Methods

### The study design of the current research

**Fig. 1:**
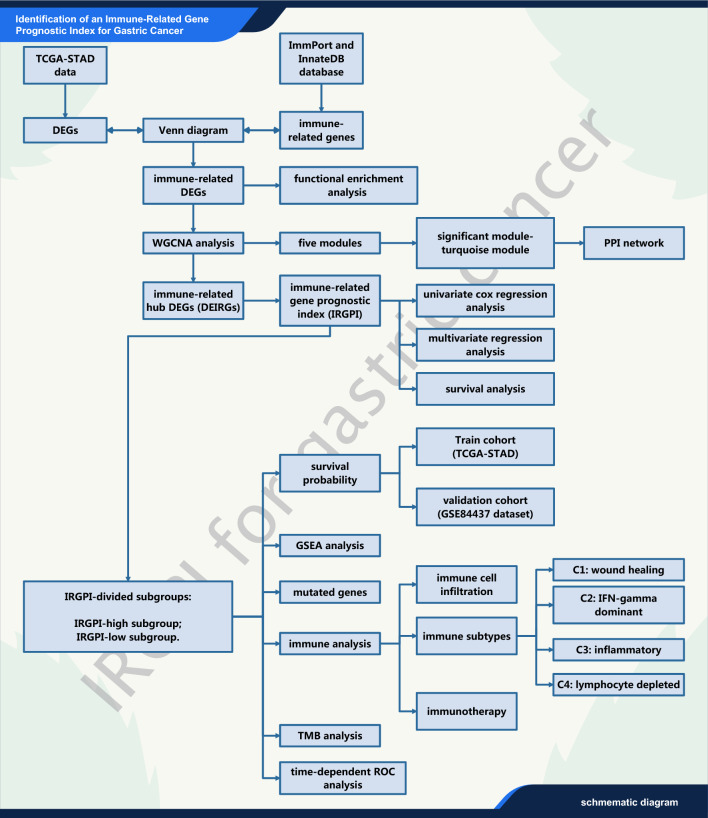
The schematic diagram of the present research

As shown in Fig. 1, this study leveraged RNA-sequencing and clinical data from TCGA and GEO databases to identify prognostic immune genes in gastric cancer. Differential expression analysis, GO and KEGG pathway enrichment, and weighted gene co-expression network analysis (WGCNA) were performed to detect immune-related hub genes. Multivariate Cox regression was utilized to develop an 8-gene immune prognostic index (IRGPI) trained on TCGA data. Samples were stratified into IRGPI-high and -low groups, and findings were validated in the GEO cohort using survival analyses. Bioinformatic approaches like GSEA, CIBERSORT, and TIDE algorithms were employed to gain biological insights into IRGPI subgroups regarding signaling pathways, immune cell landscapes, and potential immunotherapy response.

### Collection of patients and datasets

RNA sequencing (RNA-seq) data for a collection of 407 gastric samples, comprising 375 samples of STAD (Stomach adenocarcinoma) and 32 para-cancer normal samples, was acquired from The Cancer Genome Atlas (TCGA) database, alongside their accompanying clinical and pathological information. In order to validate the findings obtained by TCGA data analysis, an independent data cohort—the GSE84437 dataset (URL: https://www.ncbi.nlm.nih.gov/geo/query/acc.cgi?acc=GSE84437) was obtained from the Gene Expression Omnibus (GEO) database based on the keywords searching “gastric cancer”. The GSE84437 contained the mRNA expression profiling and the survival information of 483 gastric cancer samples. The inclusion criteria for selecting the GSE84437 dataset were listed below: (1) expression level of DUSP1Experimental type: Expression profiling by array; (2) Organism: Homo sapiens; (3) Investigated cancer type: gastric cancer; (4) With overall survival information for each sample; (5) With clinopathological information: for example, age, sex, and TNM stage; [5] The sample size of the GEO dataset should be comparable with the sample size of TCGA dataset.

Furthermore, two immune genes-related databases (i.e., ImmPort and InnateDB databases) were used to download the immune-related genes. The ImmPort database (version 49; weblink: https://www.immport.org/home) is an extensive and invaluable resource that offers researchers a comprehensive array of immunology data, encompassing experimental findings, clinical data, and an extensive repository of information on immune-related diseases, facilitating the advancement of immunological research and understanding. The InnateDB database (Verison 5.4; weblink: https://www.innatedb.com/) is an accessible database containing information on genes, proteins, experimentally confirmed interactions, and signaling pathways associated with the innate immune response in humans, mice, and bovines to microbial infection. The InnateDB database not only annotated innate immunity interactions and pathways but also annotated genes involved in the innate immune response. Moreover, gene mutation information was acquired from the cBioPortal database.

### Detection of immune-related hub genes

Differential expression analysis was conducted to identify genes deregulated between 375 stomach adenocarcinoma (STAD) tumor samples and 32 adjacent healthy tissue samples from the TCGA_STAD dataset. The limma package (version 3.18; weblink: https://bioconductor.org/packages/release/bioc/html/limma.html) in R was used to perform the differential expression analysis. Raw read counts were normalized, and then voom transformed to log2-counts per million for differential testing. Genes were called differentially expressed genes based on fold change and statistical significance thresholds. Specifically, genes with |log2(fold change)|> 1 and FDR–adjusted p-value < 0.05 were designated as differentially expressed genes (DEGs). The log2(fold change) threshold 1 indicates a minimum two-fold change in either direction. The FDR adjustment corrects p-values for multiple hypothesis testing using the Benjamini–Hochberg procedure.

Afterward, these DEGs were compared to immune-related gene lists obtained from ImmPort and InnateDB databases, and differentially expressed immune-related genes (DEIRGs) were obtained. Gene Ontology (GO) and Kyoto Encyclopedia of Genes and Genomes (KEGG) enrichment analysis of the DEIRGs was conducted using the clusterProfiler R package (version 3.18; weblink: https://bioconductor.org/packages/release/bioc/html/clusterProfiler.html), aiming to delve deeper into the immune-related functionalities of the obtained data. Hypergeometric tests were utilized to identify enriched functional categories and pathways. p-values were adjusted for multiple comparisons using the Benjamini–Hochberg procedure to control false discovery rate (FDR). An adjusted p-value threshold of 0.05 and FDR q-value < 0.25 were applied to determine the statistical significance of enriched terms. Only categories with a minimum of 10 overlapping genes were assessed to ensure adequate representation. Gene length bias was controlled by employing the clusterProfiler internal gene length bias correction.

To detect hub genes, we conducted Weighted Gene Co-expression Network Analysis (WGCNA) using the WGCNA package (version 1.72–1; weblink: https://cran.r-project.org/web/packages/WGCNA/index.html). To identify co-expressed gene modules, weighted correlation network analysis was performed. Initially, we generated a similarity matrix by calculating the Pearson correlation coefficient between pairs of genes based on their expression data. This similarity matrix was then converted into an adjacency matrix. A soft thresholding power of β = 5 was selected by analyzing the network topology at various thresholds to achieve an approximate scale-free topology fit with R^2^ > 0.8. Next, a signed hybrid weighted co-expression similarity was calculated using the power adjacency function and converted into a topological overlap matrix (TOM), which quantifies the level of association between genes. Hierarchical clustering based on TOM-based dissimilarity (1-TOM) was used to identify gene modules with high topological overlap. The genes were clustered using a distance metric of 1-TOM, and a dynamic pruning tree was constructed to determine the modules. Dynamic tree cut algorithm with a minimum module size of 30 genes and a merging height cutoff of 0.25 was applied to identify consensus modules. Ultimately, five modules were identified. For the modules with significant relevance, particularly the turquoise modules, we created a network by connecting genes with an edge weight greater than 0.2. To explore genetic alterations, we analyzed somatic mutations in the hub genes related to the immune response using the ComplexHeatmap package (version 3.18; weblink: https://bioconductor.org/packages/release/bioc/html/ComplexHeatmap.html) in R.

### Establishment of the IRGPI index

We developed an immune-related gene prognostic index (IRGPI) by leveraging the significant impact on overall survival (OS) of immune-related hub genes. This index was constructed through multivariate Cox regression analysis. To calculate the IRGPI for each sample, we multiplied the expression values of specific genes by their respective weights in the Cox model and aggregated them together. To assess the independent prognostic value of the IRGPI, univariate and multivariate Cox regression analyses were performed. The prognostic strength of the IRGPI was evaluated using Kaplan–Meier (K-M) survival curves with log-rank tests in both the TCGA and GEO cohorts.

### Gene set enrichment analysis and gene mutation analysis

To explore the signaling pathways associated with the immune-related gene prognostic index (IRGPI), we initially performed a differential expression analysis using the limma package (version 3.18; weblink: https://bioconductor.org/packages/release/bioc/html/limma.html) in R. This analysis aimed to identify genes that exhibited differential expression between samples with high and low IRGPI scores. Subsequently, we conducted enrichment analysis using the gene set enrichment analysis (GSEA) method with KEGG gene sets, implemented through the clusterProfiler package (version 3.18; weblink: https://bioconductor.org/packages/release/bioc/html/clusterProfiler.html) in R. Enriched pathways were identified using a hypergeometric test with Benjamini–Hochberg adjustment for multiple comparisons. The adjusted p-value threshold was set at 0.05, and FDR < 0.25 was considered statistically significant. A minimum gene set size of 10 was required to assess a category.

To further investigate genetic alterations within the IRGPI subgroups, we utilized data from the cBioPortal database. The Maftools package (version 3.18; weblink: https://bioconductor.org/packages/release/bioc/html/maftools.html) in R was employed to analyze the quantity and quality of gene mutations observed in these subgroups. Total mutation burden (TMB) was determined for each TCGA-STAD sample using whole exome sequencing data. The total number of nonsynonymous mutations was counted and normalized by the size of the coding region targeted for sequencing. TMB was quantified as the number of mutations per megabase (Mb). The relationship between IRGPI and TMB was examined using Pearson's correlation coefficient. A two-tailed test was performed to assess statistical significance. A p-value < 0.05 was considered significant. The correlation coefficient (r) and p-value were calculated in R using the cor.test() function.

### Immune analysis

To assess the immune cell landscape of gastric cancer samples, we utilized the CIBERSORT computational tool available at https://cibersort.stanford.edu/. This deconvolution algorithm uses gene expression data to estimate the composition of immune cells in a mixed cell population. The LM22 signature matrix containing 547 gene markers of 22 human hematopoietic cell phenotypes was applied to the gastric cancer RNA-seq data from TCGA. CIBERSORT was run using 100 permutations and quantile normalization disabled. Relative proportions of the 22 immune cell types (e.g., CD8 + T cells, neutrophils, macrophages) were computed for each sample by normalizing the deconvolved matrix using the sum method. Samples with a CIBERSORT p-value < 0.05 were considered successful deconvolution of immune cell fractions. Subsequently, we compared the relative proportions of these immune cell types and clinicopathologic factors between the two immune-related gene prognostic index (IRGPI) subgroups, visualizing the results in a landscape map.

Additionally, to further investigate differences in immune subtypes between the two IRGPI subgroups, we employed the RColorBrewer package (version 1.1–3; weblink: https://cran.r-project.org/web/packages/RColorBrewer/index.html) in R to create a heatmap and table, displaying the distribution of TCGA immune subtypes (C1: wound healing; C2: IFN-gamma dominant; C3: inflammatory; C4: lymphocyte depleted) within the IRGPI subgroups. To evaluate the potential clinical benefit of immunotherapy across IRGPI subgroups, we utilized the Tumor Immune Dysfunction and Exclusion (TIDE) algorithm (https://tide.dfci.harvard.edu/) to derive an immunotherapy response prediction score. TIDE integrates tumor mutation burden, microsatellite instability (MSI), and gene expression data to stratify patients based on the likelihood of response to immune checkpoint inhibitors. TIDE gene expression input was derived from RNA-seq data matched to each sample. No additional tuning or customization of TIDE parameters was performed. TIDE was run using default settings to generate three output scores—an immunotherapy response prediction, MSI score, and T-cell dysfunction score for each sample. TIDE output scores between IRGPI high and low subgroups were compared using Wilcoxon rank sum tests to assess immunotherapy efficacy across prognostic groups.

To determine the prognostic predictive value of IRGPI, time-dependent ROC analysis was performed using the timeROC package (version 0.4; weblink: https://cran.r-project.org/web/packages/timeROC/index.html) in R. This method assesses the discrimination ability of a biomarker by evaluating its performance in predicting the probability of an event over time. Briefly, sensitivity and specificity are calculated at each observed event time point to plot the ROC curve. The area under the time-dependent ROC curve (time-dependent AUC) measures model's prognostic accuracy over the entire follow-up period. In general, AUC values are interpreted as follows: 0.5–0.6 (failed), 0.6–0.7 (worthless), 0.7–0.8 (poor), 0.8–0.9 (good), and > 0.9 (excellent). In our analysis, AUC values were computed at discrete time points of 3 and 5 years to evaluate the prognostic performance of IRGPI at clinically meaningful horizons. Time-dependent AUC was also calculated for TIDE and TIS scores, and compared to IRGPI to determine relative prognostic utility. Sensitivity, specificity, and accuracy metrics were generated at each time point.

## Statistical analysis

To compare continuous variables between the two groups, we used an independent t-test. The chi-square test was employed to assess categorical data. TIDE scores were compared between the groups using the Wilcoxon test. For univariate survival analysis, we utilized the Kaplan–Meier method in conjunction with the log-rank test. Multivariate survival analysis was performed using the Cox regression model. A significance level of p < 0.05 was considered in all statistical tests, with significance determined by values on both sides of the distribution.

## Results

### Identification of the differentially expressed immune-related genes and their enriched functions

**Fig. 2:**
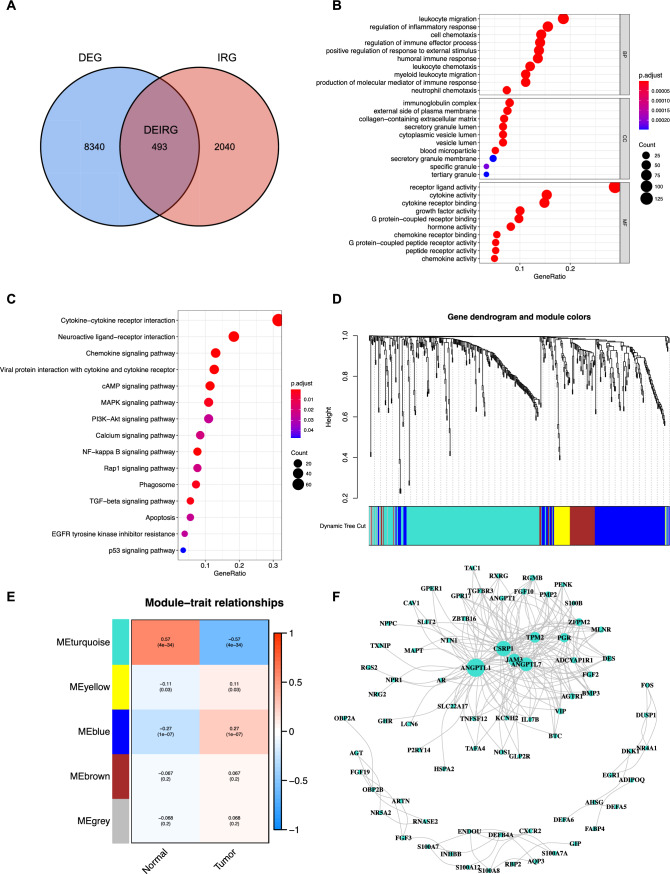
The functional enrichment analysis of DEIRGs and identification of hub DEIRGs. **A** Venn diagram shows that 493 DEIRGs were overlapped between 8833 DEGs and 2533 IRGs; **B** Gene Ontology (GO) enrichment analysis of the DEIRG (p < 0.05); **C** Kyoto Encyclopedia of Genes and Genomes (KEGG) pathway analysis of the DEIRG (p < 0.05); **D** Weighted gene coexpression network analysis (WGCNA) of immune-related differentially expressed genes with a soft threshold β = 5. Gene dendrogram representing the hierarchical clustering of genes based on their expression patterns; **E** The heatmap illustrates the module-trait relationships between the identified gene modules and trait-sample type (healthy control/tumor). Each row represents a module, and each column represents a trait. The level of correlation is indicated by color, with red colors representing positive correlations and blue colors representing negative correlations. **F** The PPI network of the genes in the turquoise module

Through overlapping 8833 DEGs and 2553 ImmPort- and InnateDB-obtained IRGs (Immune-related genes), 493 DEIRGs (differentially expressed immune related genes) were obtained (Fig. 2A). The results regarding Gene Ontology (GO) terms enriched in DEIRGs show that 493 DEIRGs were significantly enriched in several biological processes such as leukocyte migration, leukocyte chemotaxis, myeloid leukocyte migration, neutrophil chemotaxis; several cellular components for example collagen-containing extracellular matrix, immunoglobulin complex, and secretary granule membrane; several molecular functions such as cytokine activity, cytokine receptor binding, chemokine receptor binding, and chemokine activity (Fig. 2B). The KEGG enrichment analysis shows that DEIRGs were enriched in several key signaling pathways, for instance, MAPK signaling pathway, PI3K-Akt signaling pathway, NF-kappa B signaling pathway, Rap1 signaling pathway, TGF-beta signaling pathway, EGFR tyrosine kinase inhibitor resistance, and p53 signaling pathway (Fig. 2C).

### Identification of the key module by performing WGCNA analysis

Figure 2D shows that the gene dendrogram was generated using the WGCNA algorithm (Weighted Gene Co-expression Network Analysis) with a soft threshold β = 5. The module colors were assigned based on the unsupervised clustering algorithm employed by WGCNA. The module colors in the dynamic tree cut figure reflect the different co-expression modules. Notably, the turquoise and blue module represent the largest module, suggesting these two modules’ potential importance in the biological processes under investigation. Figure 2E shows the associations between gene modules (e.g., turquoise, yellow, blue, brown, and grey) and various phenotypic traits (healthy control samples/tumor samples). Positive or negative correlations indicate the potential functional relevance of specific gene modules to the corresponding traits. For instance, the blue module exhibits a significant positive correlation with Trait-tumor samples (correlation value = 0.27, p = 1e−07), suggesting its potential involvement in the biological processes underlying tumor samples. Conversely, the turquoise module with Trait-tumor samples (correlation value = – 0.57, p = 4e–34), implying a potential inhibitory or opposing relationship. Figure 2F shows the PPI network constructed by the genes in the turquoise module. The gene nodes with the highest degree were shown to be ANGPTL1, CSRP1, TPM2, PGR, ZFPM2, ANGPTL7, JAM3, MLNR, ADCYAP1R1, DES, and FGF2.

### Establishment of a prognostic index

**Fig. 3:**
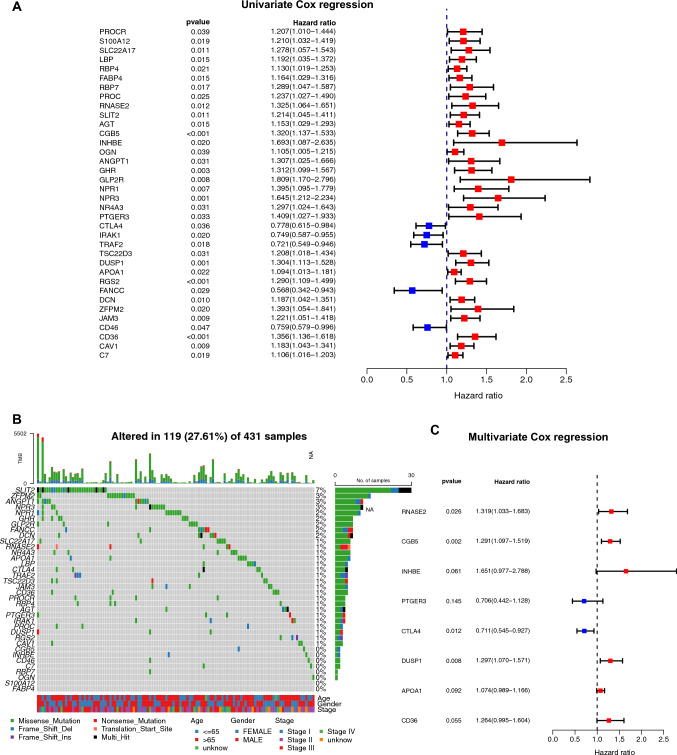
The uni- and multi-variate Cox regression analysis of DEIRGs. **A** Univariate Cox regression analysis of hub DEIRG; **B** Significantly mutated prognostic genes among the univariate Cox regression analysis-obtained statistically significant DEIRGs. Samples (columns) are arranged to emphasize mutual exclusivity among mutations. The right shows the mutation percentage, and the top shows the overall number of mutations. The color coding indicates the mutation type. **C** Multivariate Cox regression analysis of the DEIRGs which were significant in the univariate Cox analysis (p < 0.05)

The uni- and multi- variate Cox regression analyses were conducted to identify the independent prognostic DEIRGs for predicting the overall survival outcomes of STAD patients. Figure 3A shows that hub DEIRGs (e.g., PROCR (p = 0.039), S100A12 (p = 0.019), SLC22A17 (p = 0.011)) significantly affected the overall survival outcomes of STAD patients. Figure 3B shows the significantly mutated prognostic genes (e.g., SLIT2 (mutation rate = 7%), ZFPM2 (mutation rate = 3%), ANGPT1 (mutation rate = 3%), and NPR3 (mutation rate = 3%)) among the univariate Cox regression analysis-obtained statistically significant DEIRGs. The multivariate Cox regression analysis confirmed that RNASE2 (p = 0.026), CGB5 (p = 0.002), CTLA4 (p = 0.012), and DUSP1 (p = 0.008) were independent prognostic genetic factor after adjusted for other clinicopathologic factors (Fig. 3C).

Several prior studies using a similar methodology to develop prognostic gene signatures have described the thought behind choosing the Cox model for developing the IRGPI formula [13–17]. Based on the results of multivariate Cox regression analysis, a prognostic index for STAD tumor samples was established based on the formula immune-related gene prognostic index (IRGPI) = expression level of RNASE2* 0.277 + expression level of CGB5* 0.255 + expression level of INHBE*0.501 + expression level of PTGER3*(– 0.348) + expression level of CTLA4*(– 0.341) + expression level of DUSP1*0.260 + expression level of APOA1*0.071 +  + expression level of CD36*0.234.

### Comparison of the survival probability between two IRGPI-defined clusters

**Fig. 4:**
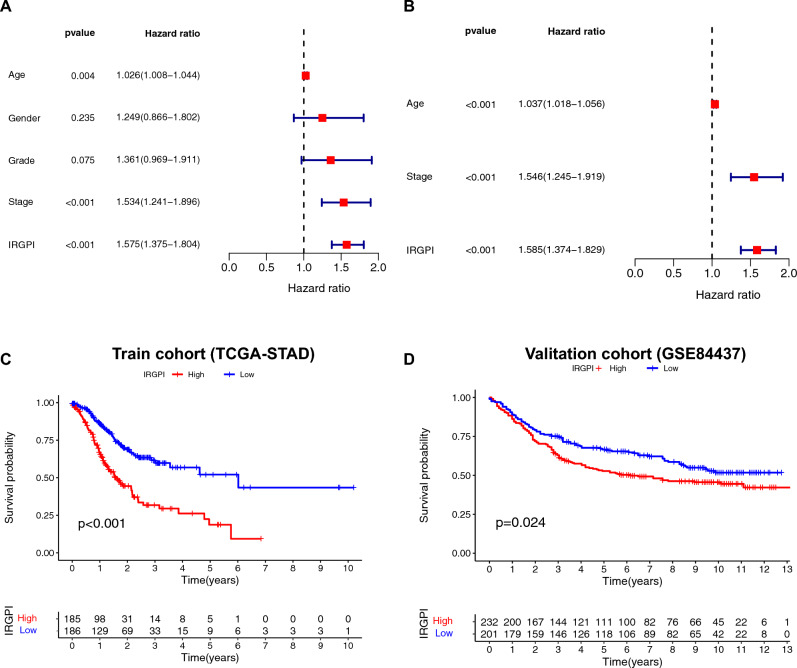
Prognostic analysis based on immune-related gene prognostic index (IRGPI). **A** Univariate Cox regression analysis of clinicopathologic factors and the IRGPI in train cohort (TCGA-STAD); **B** Multivariate Cox regression analysis of the factors significant in the univariate Cox regression analysis (p < 0.05); **C** K–M survival analysis of the IRGPI subgroups in the training cohort (TCGA-STAD); **D** K–Msurvival analysis of the IRGPI subgroups in the validation cohort (GSE84437)

Figure 4A, B shows that univariate and multivariate Cox regression analysis confirmed that IRGPI genes were independent prognostic factors (p < 0.001) after adjusting for clinicopathologic factors including age, gender, grade, and stage. The cutoff to categorize STAD samples into IRGPI-high and low groups was determined by taking the median value of the IRGPI risk scores as the threshold. The Kaplan–Meier survival analysis based on the training cohort dataset (TCGA-STAD) shows that the patients in the IRGPI-high expression cluster indicated lower survival probability and thus worse prognosis; while the patients in the IRGPI-low expression cluster represented higher survival probability and thus favorable prognosis (p < 0.001, Fig. 4C). The results validated by using the GSE84437 as the validation cohort dataset obtained the same trend by showing a higher survival probability in the IRGPI-low expression STAD patients compared with the IRGPI-high expression STAD patients (p−0.024 < 0.05, Fig. 4D).

### Identification of the biological functions of IRGPI-based subgroups

**Fig. 5:**
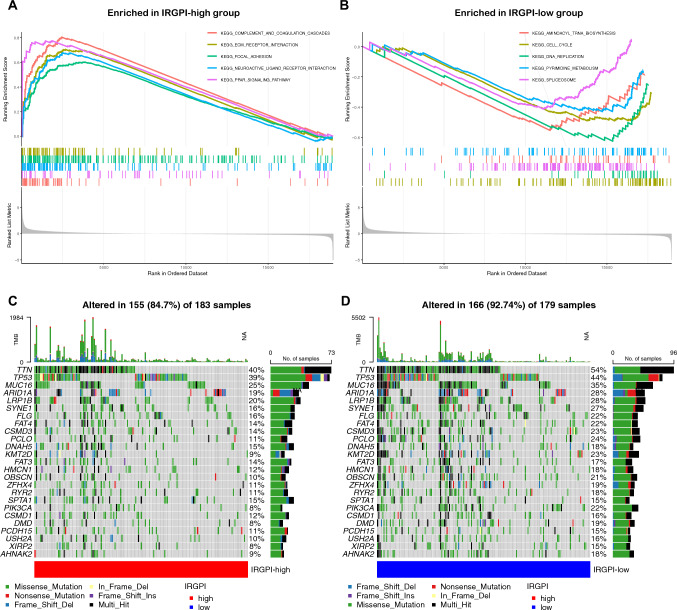
Molecular characteristics of different IRGPI subgroups. **A** Gene set enrichment analysis in IRGPI-high subgroup (p < 0.05, FDR < 0.25); **B** Gene set enrichment analysis in IRGPI-low subgroup (p < 0.05, FDR < 0.25); **C, D** Significantly mutated genes in the mutated GC samples of IRGPI-high **(C)** and IRGPI-low **(D)** subgroups

GSEA was performed to determine the gene sets enriched in two IRGPI subgroups, including IRGPI-high and IRGPI-low subgroups. The gene sets of the IRGPI-high samples were enriched in complement and coagulation cascades, ECM receptor interaction, focal adhesion, neuroactive ligand receptor interaction, and PPAR signaling pathway (Fig. 5A). The gene sets of the IRGPI-low samples were enriched in Aminoacyl-TRNA-biosynthesis, cell cycle, DNA replication, pyrimidine metabolism, and spliceosome (Fig. 5B). Afterward, the gene mutations were analyzed to identify the significantly mutated genes in the mutated STAD samples of two different IRGPI-based subgroups. Figure 5C, D shows that the top 10 genes with the highest mutation rates in both the IRGPI-high and -low subgroups was identified to be TNN, TP53, MUC16, ARID1A, LRP1B, SYNE1, FLG, FAT4, CSMD3, and PCLO.

### Immune characteristics of two different IRGPI subgroups

**Fig. 6:**
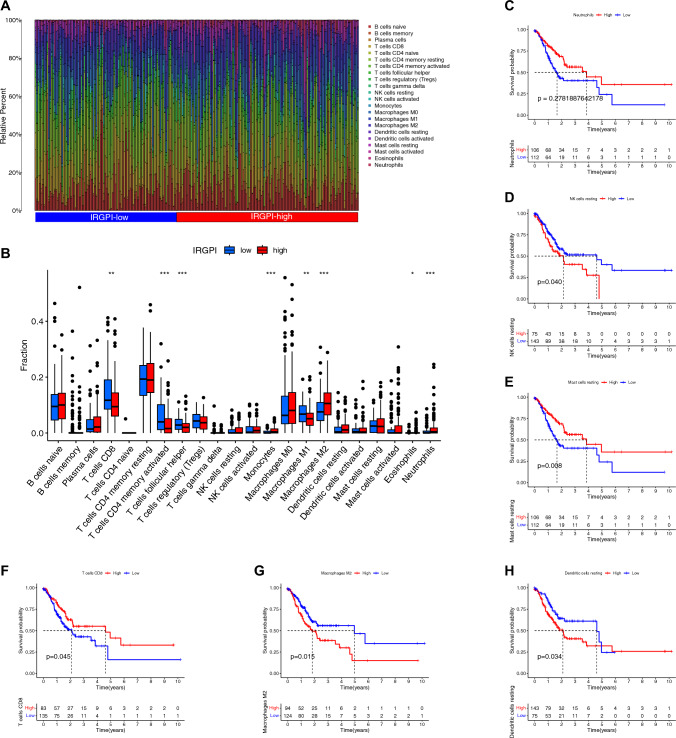
The characteristics of immune cell infiltration in different IRGPI subgroups. **A** The IRGPI grouping and proportions of immune cells for GC patients in TCGA cohort. **B** The fractions of TME cells in different IRGPI subgroups. The scattered dots represent the immune score of the two subgroups. The thick lines represent the median value. The bottom and top of the boxes are the 25th and 75th percentiles (interquartile range), respectively. Significant statistical differences between the two subgroups were assessed using the Wilcoxon test (*p < 0.05; **p < 0.01;***p < 0.001). **C–H** KM plots of six types of immune cells (neutrophils **(C)**, NK cells resting **(D)**, mast cells resting **(E),** T cells resting **(F)**, macrophage M2 **(G)**, dendritic cells resting (**H**).

The Wilcoxon test was utilized to compare the relative percentage of immune cells in different IRGPI subgroups (Fig. 6A). Figure 6B shows that monocytes, macrophage M2, and neutrophils were more abundant in the IRGPI-high subgroup. A predominant M2 macrophage population may suppress anti-tumor immune responses and promote a favorable environment for tumor growth. In the IRGPI-low subgroup, T cells CD8, T cells CD4 memory activated, T cells follicular helper, and macrophage M1 were more abundant. An increased presence of M1 macrophages can drive an effective anti-tumor immune response. Such findings indicate that the IRGPI-high subgroup is more aggressive while the IRGPI-low subgroup is less aggressive.

In addition, Kaplan–Meier plot analysis was performed to investigate the prognostic values of these significantly abundant immune cells. Figure 6C shows that the score of neutrophils didn’t show significant prognostic values in STAD patients. Figure 6D shows that STAD patients with a low score of NK cells resting had a favorable overall survival outcome, while STAD patients with a higher score of neutrophils had a worse prognosis (p = 0.040 < 0.05). Figure 6E shows that STAD patients with a high score of mast cells resting had a favorable overall survival outcome, while STAD patients with a low score of mast cells resting had a worse prognosis (p = 0.008 < 0.05). Figure 6F shows that STAD patients with a high score of Tcells CD8 had a favorable overall survival outcome, while STAD patients with a low score of T cells CD8 had a worse prognosis (p = 0.045 < 0.05). Figure 6G shows that STAD patients with a low score of macrophage M2 had a favorable overall survival outcome, while STAD patients with a higher score of macrophage M2 had a worse prognosis (p = 0.015 < 0.05). Figure 6H shows that STAD patients with a low score of dendritic cells resting had a favorable overall survival outcome, while STAD patients with a higher score of dendritic cells resting had a worse prognosis (p = 0.034 < 0.05).

**Fig. 7:**
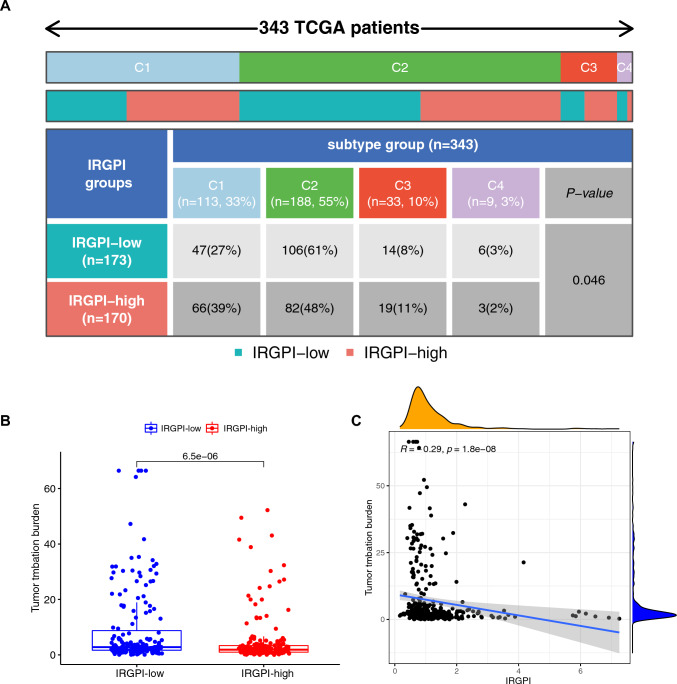
Distribution of immune subtypes in different IRGPI subgroups. **A** Heatmap and table showing the distribution of immune subtypes (C1: wound healing; C2: IFN-gamma dominant; C3: inflammatory; C4: lymphocyte depleted) between the IRGPI subgroups; **B** The difference of total mutational burden between IRGPI subgroups; **C** Correlation analysis between IRGPI and total mutational burden

Figure 7A shows the distribution of immune subtypes (C1: wound healing; C2: IFN-gamma dominant; C3: inflammatory; C4: lymphocyte depleted) between the two IRGPI-based subgroups. There were more wound healing samples and inflammatory samples in the IRGPI-high subgroup compared to the IRGPI-low subgroup; more IFN-gamma dominant samples and lymphocyte depleted samples in the IRGPI-low subgroup compared than the IRGPI-low subgroup (p = 0.046 < 0.05). Figure 7B shows that the TMB was significantly higher in the IRGPI-low subgroup compared to the IRGPI-high subgroup (p = 6.5e−06). Figure 7C shows a negative correlation between IRGPI expression and TMB (r = − 0.29, p = 1.8e−08).

**Fig. 8:**
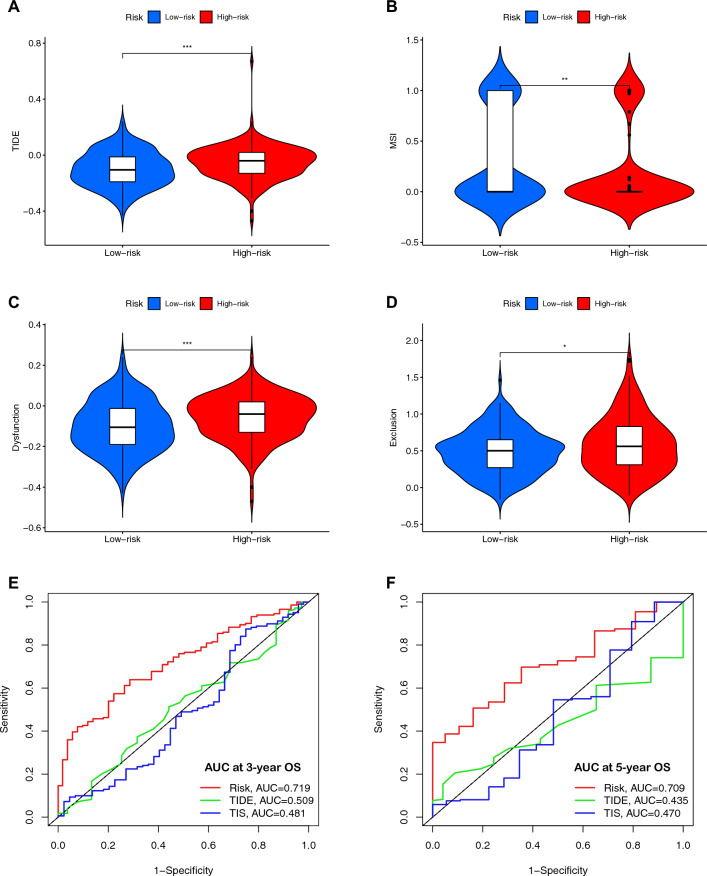
The potential clinical efficacy of immunotherapy in different IRGPI subgroups and the value of predicting the prognosis of IRGPI in patients with GC. **A–D** Tumor immune dysfunction and exclusion (TIDE) **(A)**, microsatellite instability (MSI) **(B)**, and T-cell dysfunction **(C)** and exclusion score **(D)** in different IRGPI subgroups. The scores between the two IRGPI subgroups were compared through the Wilcoxon test (*p < 0.05; **p < 0.01; ***p < 0.001). **E** ROC analysis of IRGPI, TIS, and TIDE on OS at 3-years follow-up in TCGA cohort. **F** ROC analysis of IRGPI, TIS, and TIDE on OS at 5-years follow-up in TCGA cohort

The various immune-related scores (e.g., Tumor immune dysfunction and exclusion (TIDE) (Fig. 8A), microsatellite instability (MSI) (Fig. 8B), T-cell dysfunction (Fig. 8C), and exclusion score (Fig. 8D)) were compared between two IRGPI subgroups. Figure 8E, F shows that IRGPI had good diagnostic values in predicting the overall survival at 3-year (AUC = 0.719) and 5-year post operation (AUC = 0.709).

## Discussion

The IRGPI index developed in the current study comprised eight genes: RNASE2, CGB5, INHBE, PTGER3, CTLA4, DUSP1, APOA1, and CD36. RNASE2, also known as Ribonuclease A family member 2, has been found to regulate the immune response by modulating the activity of macrophages and lymphocytes [18]. RNASE2 expression is associated with the activation of M2 macrophages, which secrete immunosuppressive factors that dampen the antitumor immune response and further promote tumor growth and immune suppression [19]. RNASE2 has been shown to regulate the production of cytokines (e.g., Interleukin-4 (IL-4) and Interleukin-10 (IL-10)), which can modulate the immune response and promote an immunosuppressive microenvironment, allowing tumor cells to escape immune surveillance [20]. INHBE (inhibin beta E subunit) is upregulated in gastric cancer and may contribute to immune dysfunction by suppressing T cell activity [21]. INHBE expression is correlated with increased angiogenesis and the production of pro-inflammatory molecules in gastric cancer [22]. PTGER3 is a receptor for prostaglandin E2 (PGE2), a lipid mediator that can promote immunosuppression [23]. PGE2 binding to PTGER3 on immune cells can inhibit various immune functions, including T cell activation, proliferation, and cytokine production [24]. PGE2-PTGER3 signaling has been shown to mediate the polarization of TAMs towards a pro-tumor phenotype, and promote the production of immunosuppressive factors (TGF-β (transforming growth factor-beta) and IL-10 (interleukin-10)) [25]. CTLA4 (Cytotoxic T Lymphocyte Antigen 4) is an immune checkpoint molecule primarily expressed on T cells, especially regulatory T cells (Tregs) [26]. This interaction between CTLA4 on Tregs and tumor cell ligands inhibits the activation of effector T cells and promotes immune evasion in gastric cancer [27]. DUSP1 (Dual-specificity phosphatase 1), also known as MAP kinase phosphatase 1 (MKP-1), is a protein phosphatase involved in regulating the mitogen-activated protein kinase (MAPK) signaling pathway [28]. Higher expression of DUSP1 has been linked to better overall survival, indicating its potential as a prognostic marker in gastric cancer [29]. DUSP1 can exert tumor-suppressive effects in gastric cancer by negatively regulating MAPK signaling pathways that control cell proliferation, survival, and invasion [30]. As the primary protein component of high-density lipoproteins (HDL), apolipoprotein A1 (APOA1) levels may enable better prediction of an individual's cancer risk, earlier diagnosis of malignancy, tracking of disease progression during follow-up, and stratification of patients based on likely prognosis [31]. APOA1 was identified at decreased levels in plasma from mice bearing larger gastric tumor xenografts compared to those with smaller tumors [32]. A low level of plasma APOA1 was found to have potential utility as a blood-based biomarker for detecting gastric cancer progression and distinguishing malignant from benign conditions [32]. The underlying mechanim by which ApoA-I mimetic peptides influence antitumor immunity involves the elevation of certain oxidized lipids, which then activate Notch signaling [33]. The stimulation of the Notch pathway, in turn, results in an increased population of patrolling monocytes [33]. CD36 (Cluster of Differentiation 36) is a cell surface receptor involved in lipid metabolism, inflammation, and immune modulation [34]. CD36 plays a role in the polarization and function of tumor-associated macrophages (TAMs), which has been associated with a pro-inflammatory phenotype and increased tumor-promoting activities [35].

Figure 3C, D shows that the survival probability in the IRGPI-low expression STAD patients was significantly higher than that in the IRGPI-high expression STAD patients. This observation can be due to the findings that monocytes, macrophage M2, and neutrophils in the IRGPI-high subgroup were more abundant compared to that in the IRGPI-low subgroup; while T cells CD8, T cells CD4 memory activated, T cells follicular helper, and macrophage M1 in the IRGPI-low subgroup were more abundant compared to that in the IRGPI-high subgroup. The abundant presence of M2 macrophages in the tumor microenvironment has been suggested to be associated with worse prognosis in cancer patients due to their tumor-promoting functions, immunosuppressive effects, pro-metastatic activities, and resistance to therapy [36]. M2 macrophages have immunosuppressive properties and inhibit effective anti-tumor immune responses, which are manifested by inhibiting T cell activation and proliferation, impairing natural killer (NK) cell function, and promoting the expansion of regulatory T cells (Tregs) [37]. By contrast, a higher proportion of M1 macrophages in the tumor microenvironment is generally considered beneficial for cancer patients, as it promotes anti-tumor immune responses and improves survival outcomes [38]. M1 macrophages produce various pro-inflammatory cytokines, such as tumor necrosis factor-alpha (TNF-α), interleukin-1 beta (IL-1β), and interleukin-6 (IL-6), which have been shown to inhibit tumor growth [39]. Additionally, M1 macrophages have high antigen-presenting capacity, so they can efficiently present tumor-specific antigens to T cells, leading to an enhanced anti-tumor immune response [40].

It is important to acknowledge the strengths and limitations in this current research. The strength regarding the study design is the utilization of WGCNA analysis. WGCNA is a systems biology methodology utilized for the construction of gene co-expression networks and the identification of functionally relevant gene modules and hub genes [41]. By taking into account the intricate co-expression and interactions among genes, WGCNA discerns the hub genes with respect to functionalities from a holistic network perspective, rather than solely focusing on individual genes with differential expressions [41].WGCNA offers many obvious advantages in identifying hub genes: It captures complex gene–gene interactions, provides a holistic view of gene networks; it prioritizes central hub genes with significant functional relevance; it handles large-scale datasets and allows for the integration of different types of data [42]. Additionally, it is worthwhile to emphasize the potential limitation of the present research. Firstly, the study relied solely on RNA sequencing data, which provides information about gene expression levels but does not capture post-translational modifications or protein–protein interactions. Secondly, the study used a relatively simplified approach to construct the immune-related gene prognostic index (IRGPI) by multiplying gene expression values with their weights in the Cox model. This approach assumes a linear relationship between gene expression levels and patient survival, which may oversimplify the underlying biological complexity. Furthermore, the study did not explicitly investigate the functional mechanisms through which the identified immune-related hub genes influence the prognosis of gastric cancer patients. Experimental validation and functional studies are needed to elucidate the underlying molecular pathways and mechanisms. Overall, while the study provides valuable insights into the immune-related gene expression and prognosis of gastric cancer patients, these limitations should be when interpreting the results. Further studies with larger sample sizes, experimental validations, and comprehensive consideration of confounding factors are needed to strengthen the findings.

The findings of this study have important implications for future research on gastric cancer and immune-related gene expression. Firstly, the identified immune-related hub genes and the constructed immune-related gene prognostic index (IRGPI) provide a foundation for further investigations into the immunological landscape of gastric cancer. The IRGPI and related pathway and functional analysis results can be applied for risk stratification and personalized follow-up strategies, inform potential novel immune therapeutic targets, and aid in predicting patient responses to immune checkpoint inhibitor treatment. The differences in immune microenvironments and mutation patterns between IRGPI subgroups can also guide customized combinatorial immunotherapeutic design. Future studies can validate and expand upon these findings using larger and independent cohorts, incorporating comprehensive clinical and molecular information to strengthen the association between immune-related genes and patient prognosis. Additionally, experimental validation and functional studies should be conducted to unravel the underlying mechanisms through which these immune-related genes impact the progression and response to treatment in gastric cancer. Furthermore, considering the complex interplay between the immune system, tumor heterogeneity, and microenvironment, future research should aim to integrate multi-omics data and develop more sophisticated models to fully capture the intricate dynamics and interactions that influence the immune response and prognosis in gastric cancer. Ultimately, understanding the immune-related gene expression patterns and their clinical implications will pave the way for developing novel therapeutic strategies, including immunotherapies, for the management of gastric cancer patients.

## Conclusion

In conclusion, this study identified immune-related hub genes and developed an immune-related gene prognostic index (IRGPI) for gastric cancer based on RNA sequencing data. The findings highlight the potential importance of immune-related gene expression in predicting prognosis in gastric cancer patients. The study provides valuable insights into the immunological landscape of gastric cancer and opens avenues for further research to validate and expand upon these findings. These findings have implications for developing of novel therapeutic strategies and personalized medicine approaches in the managing of gastric cancer patients.

## Data Availability

The data analyzed during the current study are available in TCGA and GEO database with the accession numbers TCGA-STAD and GSE84437. The original contributions presented in the study are included in the article; further inquiries can be directed to the corresponding author.
